# Supplementary material for: Recommendations from Latinx Trans and Non-Binary Individuals to Promote Cancer Prevention in Puerto Rico and Florida
Source: Int J Environ Res Public Health. 2023 Jan 10;20(2):1213. doi: 10.3390/ijerph20021213 (PMC9859014; doi:10.3390/ijerph20021213)
Supplement: Supplementary file 1 [file ijerph-20-01213-s001.zip › ijerph-2035047-supplementary.pdf]

Focus Group Guide

*This guide was developed in Spanish. See below

1. **Recommendations for potential content for the development of an intervention that promotes screening for breast/cervical cancer.**
   a. What issues related to a trans person's gender identity should be included in an intervention to promote cancer prevention? (If not understood, provide examples or simplify)
   b. What topics should be included in an intervention to promote doctor visits for routine cancer screenings?
   c. What topics should be included in an intervention to promote self-screening of B/C cancer? (EJ: Sensitivity and empathy with the patient cultural humility, trans-specific self-care, and how these are demonstrated in the treatment at the time of the medical visit)

2. **Specific strategies to encourage trans people to get screened for cancer.**
   a. What factors do you think can discourage (or be an institutional/medical barrier, at the time of...) a trans person getting regularly screened for cancer?
   b. What recommendations do you have that can motivate or facilitate a trans person to get cancer screenings?

3. **Expectations about sensitive treatment in medical examination procedures with this population.**
   a. What elements should be part of a trans-affirmative approach during the medical encounter and physical examination processes related to cancer screening? Please share an example.
   b. What factors might keep a trans person from getting screened for cancer? (EX: Refusing to return prior to an in-person medical appointment or initial online/phone screening)

4. **Preferred format or medium for the implementation of the intervention, including ideas for incorporating the internet, cell phones, apps, and any other technology and social media.**
   a. Taking into consideration the social techniques most commonly used by the trans community in RP (e.g. cell phones, smartphones, Facebook and Twitter), which do you think would be most appropriate to use in an intervention to promote cancer screening? (Or the most accessible in your opinion) And why? (What do you think of the idea of podcasts?)
   b. What recommendations do you have for integrating accessible technology into cancer screening? (Do there are any concerns about this use?)

End:

We have noticed that there is resistance on the part of some sectors of the community to participate in the investigations:

a. Why do you think it is?
b. How can we overcome that resistance? (what we should do, how we take care of it, how we gain the trust of the community)
c. Do you think the community would be interested in participating in another study to see the effectiveness of an intervention to promote cancer prevention? (Why yes or no; how to approach them)

Anything you want to add that we haven't asked?

Once again, we thank you very much for your time and for all the valuable contributions in the group.

Guía de grupo focal
[Spanish Version]

5. **Recomendaciones para el contenido potencial para el desarrollo de una intervención que promueva el examen de cáncer de seno/cervical.**
    d. ¿Qué temas relacionados a la identidad de género de una persona trans deben ser incluidos en una intervención para promover la prevención de cáncer? (Si no se comprende, proveer ejemplos o simplificar)
    e. ¿Qué temas deben ser incluidos en una intervención para promover las visitas al médico para exámenes rutinarios de cáncer?
    f. ¿Qué temas deben ser incluidos en una intervención para promover la auto-examinación del cáncer S/C? (EJ: Sensibilidad y empatía con el pt. humildad cultural, auto-cuidado trans-especifico, y como estos se demuestran en el trato al momento de la visita médica)

6. **Estrategias específicas para promover que las personas trans se realicen exámenes de cáncer.**
    a. ¿Qué factores crees que pueden desmotivar (o ser una barrera institucional / medica, al momento de…) una persona trans realizarse regularmente exámenes de cáncer?
    b. ¿Qué recomendaciones tienes que puedan motivar o facilitar que una persona trans se realice exámenes de cáncer?

7. **Expectativas sobre el trato sensible en los procedimientos de examinación médica con esta población.**
    a. ¿Qué elementos deben ser parte de un enfoque trans-afirmativo durante el encuentro médico y los procesos de examinación física relacionados a la examinación del cáncer? Por favor, comparta un ejemplo.
    b. ¿qué factores podrían alejar a una persona trans de hacer un examen de cáncer? (EJ: Negarse a regresar previo a una cita médica presencial o un cernimiento inicial en línea/teléfono)

8. **Formato o medio preferido para la implantación de la intervención, incluyendo ideas para incorporar el internet, teléfonos celulares, apps, y cualquier otra tecnología y medio social.**
   a. Tomando en consideración las técnicas sociales más comúnmente utilizadas por la comunidad de personas trans en PR (Ej. Celulares, teléfonos inteligentes, Facebook y Twitter), ¿cuáles creen que serían las más apropiada para utilizar en una intervención para promover los exámenes de cáncer? (O las más accesibles en tu opinión) Y ¿Por qué? (¿Qué les parece la idea de podcasts?)
   b. ¿Qué recomendaciones tienes para integrar la tecnología accesible a la examinación de cáncer? (¿Surgen algunas preocupaciones sobre este uso?)

Finalizar:
Hemos notado que existe una resistencia por parte de algunos sectores de la comunidad para participar en las investigaciones:

   d. ¿a qué ustedes creen que se deba?
   e. ¿cómo podemos sobrepasar esa resistencia? (qué debemos hacer, cómo lo atendemos, cómo obtenemos la confianza de la comunidad)
   f. ¿creen que la comunidad estaría interesada en participar de otro estudio para ver la efectividad de una intervención para promover la prevención del cáncer? (Porqué sí o no; cómo hacerles el acercamiento)

¿Algo que quieran añadir que no hayamos preguntado?

Nuevamente les agradecemos mucho por su tiempo y por todas las valiosas aportaciones en el grupo.
